# A Review on the Effects of Supercritical Carbon Dioxide on Enzyme Activity

**DOI:** 10.3390/ijms11010233

**Published:** 2010-01-19

**Authors:** Zdeněk Wimmer, Marie Zarevúcka

**Affiliations:** 1 Institute of Experimental Botany AS CR, Isotope Laboratory, Vídeňská 1083, 142 20 Prague 4–Krč, Czech Republic; E-Mail: wimmer@biomed.cas.cz; 2 Institute of Organic Chemistry and Biochemistry AS CR, Flemingovo náměstí 2, 166 10 Prague 6–Dejvice, Czech Republic

**Keywords:** enzyme, supercritical carbon dioxide, synthesis, hydrolysis, inactivation

## Abstract

Different types of enzymes such as lipases, several phosphatases, dehydrogenases, oxidases, amylases and others are well suited for the reactions in SC-CO_2_. The stability and the activity of enzymes exposed to carbon dioxide under high pressure depend on enzyme species, water content in the solution and on the pressure and temperature of the reaction system. The three-dimensional structure of enzymes may be significantly altered under extreme conditions, causing their denaturation and consequent loss of activity. If the conditions are less adverse, the protein structure may be largely retained. Minor structural changes may induce an alternative active protein state with altered enzyme activity, specificity and stability.

## 1. Introduction

The finding that some enzymes, such as proteases, lipases, peroxidases, and esterases, are stable and active in organic solvents, has broadened immensely the scope of their applications as highly enantioselective catalysts in organic synthesis. Water-insoluble substrates can thus be transformed by enzymes in non-aqueous media [1–3], opening up new synthetic routes to pharmaceuticals. These media allow the tuning of enzyme selectivity, a progress termed media-engineering. However, protection of the environment calls for a reduction in the use of organic solvents in chemical processes, as they are volatile organic compounds, a major class of air pollutants. Therefore, processes that avoid the use of organic solvents are viewed as a valuable contribution towards more environmentally friendly chemistry. Supercritical carbon dioxide (SC-CO_2_) is often discussed as an alternative, environmentally benign reaction medium for chemical synthesis, due to its non-toxic and nonflammable nature, and its relatively low critical pressure and temperature (7.36 MPa, 31.0 °C) [4–7] which allows preservation of thermally unstable compounds. Furthermore, SC-CO_2_ is climate neutral and inexpensive because the CO_2_ used is a byproduct of industrial processes. Generally, supercritical fluids differ from ordinary solvents in having both liquid-like solubilizing power, and gas-like high diffusivities and low viscosities. These properties can be controlled by the manipulation of the temperature and pressure. Near the critical point, small changes in temperature or pressure lead to significant changes in density and density-dependent solvent properties such as the solubility parameter, the partition coefficient and the dielectric constant [8].

## 2. Inactivation of Enzymes by SC-CO_2_

Non-thermal processes for the effective sterilization and inactivation of microorganisms are currently receiving a great deal of attention in the food, pharmaceutical and other relevant industries. SC-CO_2_ treatment is an alternative microbial inactivation method that can be safely used in foods and bioactive materials at relatively low temperatures. SC-CO_2_ treatment is a non-thermal process capable of inactivating microorganisms at relatively moderate pressures such as 7.3–50.0 MPa [9–12]. When SC-CO_2_ is applied to microorganisms, microbial inactivation is affected by the effect of pressure, temperature, and exposure time [13]. In general, microbial inactivation is accelerated with increasing SC-CO_2_ pressure or temperature. This is because a higher pressure enhances CO_2_ solubility, facilitating both acidification and cellular contact and the CO_2_ also exhibits a higher solvating power [14]. Higher temperatures stimulate the CO_2_ diffusivity, and can increase the cell membrane fluidity to enhance penetration [14,15]. Generally, treating microorganisms with SC-CO_2_ at higher pressure or temperature, or treating them for a longer time results in greater microbial reductions than a treatment at lower pressure or temperature, or treating them for shorter times [16].

This method has received increasing attention since SC-CO_2_ was shown to be effective for the treatment of *Escherichia coli* [17]. Beyond the critical point of CO_2_, the difference between liquid and gas CO_2_ no longer exists in the newly formed supercritical fluid phase, in which its viscosity is lower than in the liquid state and its density and dissolving power are higher than in the gaseous state [18]. Therefore, the use of SC-CO_2_ for sterilization is considered to be more effective than the use of CO_2_ in its subcritical state. Several studies have evaluated the inactivation of *S. enterica* serotype Typhimurium using subcritical- or supercritical CO_2_ [19–21]. The *S. enterica* serotype Typhimurium was effectively inactivated by treatment with SC-CO_2_ and the authors demonstrated the impact of SC-CO_2_ treatment on cellular components such as proteins (including enzymes) and nucleic acids, as well as on cellular morphology [21]. To date, the effects of SC-CO_2_ on cell viability have been determined using traditional methods such as plating. However, plating is a retrospective and timeand labor-consuming method of determining viability that is also not always capable of providing a precise assessment of cell physiology. For example, viable but non-cultivable or partially damaged cells cannot be detected using traditional plating techniques. Furthermore, the investigative tools used to identify the mechanism of microbial inactivation by SC-CO_2_ treatment have primarily been limited to electron microscopy, UV spectrophotometry and analysis of the enzyme activity [9,12,22].

Choi *at al*. conducted flow cytometry studies to evaluate if this method could be used to provide a rapid or precise assessment and interpretation of the cell physiological states of *S. enterica* serotype Typhimurium following treatment with SC-CO_2_ [23]. Balaban *et al*. [24] used SC-CO_2_ to inactivate pectinesterase in orange juice, and Chen *et al*. [25] tried to inactive polyphenol oxidase with high pressure CO_2_. Erkmen [19,26–29] reported the inactivation of *Escherichia coli*, *Listeria monocytogenes*, *Brochothrix thermosphacta*, *Salmonella typhimurium*, and *Enterococcus faecalis* in broth and food materials by high pressure CO_2_. *Lactobacillus plantarum* was inactivated by high pressure CO_2_ and *Bacillus cereus*, *Listeria innocua*, *Staphylococcus aureus*, *Salmonella salford*, *Proteus vulgaris*, *Legionella dunnifii*, *Pseudomonas aeruginosa*, and *Escherichia coli* [30] were inactivated by near-critical CO_2_ and SC-CO_2_. However, SC-CO_2_ was considered to be difficult to apply to liquid materials because SC-CO_2_ was hard to diffuse in liquids and long periods of time were needed to obtain a sufficient inactivation effect.

Ishikawa *et al*. [31–33] established a new method using SC-CO_2_ micro-bubbles. This method improved the diffusibility by subjecting liquid materials to micro-bubbles of SC-CO_2_, which enabled the control of microorganisms and enzymes in liquid materials at lower temperatures, compared with a conventional heating method. The inactivation of *Bacillus spores*, acid protease, alcaline protease, glucoamylase, lipase, and pectinesterase was reported by using a batch method with SC-CO_2_ micro-bubbles. An apparatus for a continuous method using micro-bubbles of SC-CO_2_ was designed and constructed for application in the food industry as well. Balaban *et al*. [24] stated that enzyme inactivation with SC-CO_2_ could be predominantly attributed to the pH-lowering effect during treatment.

The continuous method with SC-CO_2_ micro-bubbles [34] was applied to enzyme inactivation in a buffer system, which had a buffer capacity similar to that of commercial liquid foods (natural juices, fruit drinks, Japanese sake). By continuous treatment with SC-CO_2_ micro-bubbles, α-amylase and acid protease were effectively inactivated rather than by a heat treatment. Compared with the inactivation efficiencies of these enzymes in deionized water by continuous treatment with micro-bubbles of SC-CO_2_, Yoshimura *et al*. [34] published that inactivation of α-amylase was slightly lower, but that of acid protease was not achieved in the buffer system. These results suggest that this method enables the effective inactivation of enzymes, although inactivation efficiency by this method was affected by initial pH and the buffer action of samples.

In the continuous system, new factors affecting the inactivation efficiency of SC-CO_2_ micro-bubble treatment appeared [35]. Dissolved CO_2_ concentration, which played an important role for the enzyme inactivation by micro-bubbles SC-CO_2_ treatment, was enhanced depending on CO_2_ flow rate. Also, it was observed that the pH of deionized water was lowered to below 3 during the treatment. In another paper, Yoshimura *et al*. [35] stated that α-amylase, which was a thermoresistant enzyme, was easily inactivated at low CO_2_ flow rate because of a temporary lowering of pH. On the other hand, inactivation efficiency of acid protease, which was acid-resistant enzyme was increased depending on a CO_2_ flow rate.

Enzyme inactivation using a new apparatus for continuous treatment with SC-CO_2_ micro-bubbles was investigated by Yoshimura *et al*. [36]. The decimal reduction time (*D* value) of α-amylase (5.0 ± 1.2 min) subjected to micro-bubbles of the SC-CO_2_ treatment (micro-bubbles-SCT) at 35 °C and 30 MPa was lower than that (227 ± 15.9 min) subjected to a heat treatment at 70 °C. *D* values are defined as the treatment time needed for 90% inactivation of initial activity at a given condition. The *D* value of acid protease was reduced by the micro-bubbles-SCT at 50 °C and 30 MPa (15.4 ± 4.1 min), compared to a heat treatment at 50 °C (233 ± 15.2 min). The activation energy for the inactivation of acid protease (135 ± 8.3 kJ mol^−1^) by the micro-bubbles-SCT was roughly one half of that (259 ± 9.0 kJ mol^−1^) of a heat treatment. These results indicated that a continuous treatment with micro-bubbles of SC-CO_2_ was more effective for enzyme inactivation.

The activity of oxidoreductases containing metals, e.g., catalase, peroxidase and polyphenol oxidase reduced by SC-CO_2_ (20 MPa and 35 °C) was also studied [37]. The enzyme activities were reduced with increasing time and pressure during the SC-CO_2_ treatment. However, lipase and pyruvate decarboxylase hardly lost any of their activities when treated with SC-CO_2_. These results showed that the possibility of a significant difference in the sensitivity of enzymes to SC-CO_2_ has to be considered at any time.

## 3. Enhancement of Enzyme Activity by Treatment of SC-CO_2_

The stability and the activity of enzymes exposed to carbon dioxide under high pressure depend on enzyme species, water content in the solution and on the pressure and the temperature in the reaction system. The most important factor that may cause enzyme activity loss is probably the depressurization step; in a long-term enzyme application its activity decreases with increasing number of depressurizations [38].

An increase in enzyme activity in the SC-CO_2_ medium is observed less frequently: the activity of crude α-amylase mixed with *Escherichia coli* or *Saccharomyces cerevisiae* increased by 21% and 35% during 2 h of a sterilization step at 20 MPa and 35 °C [39]. The stability of enzymes under high pressure and the effect of the medium on enzyme activity are interesting both for theoretical and for practical applications. The three-dimensional structure of enzymes may be significantly altered under extreme conditions, causing their denaturation and a consequent loss of their activity. If the conditions are less adverse, the protein structure may largely be retained. Minor structural changes may induce an alternative active protein state with altered enzyme activity, specificity and stability [40]. Zagrobelny and Bright [41] showed by fluorescence spectroscopic studies with trypsin that the changes in protein conformation were caused by pressurization and depressurization steps, unavoidable in a high-pressure batch system. Contrary to batch reactors, continuous-flow reactors employing SC-CO_2_ do not require depressurization to introduce substrates into the reactor or to recover products. Another advantage of the continuous-flow reactors over the batch ones results from their better process economy [42].

The effect of enzyme incubation in SC-CO_2_ on its activity at ambient pressure has been examined only in a few recent studies. Giessauf and Gamse [43] reported a several-fold increase in the activity of lipase from porcine pancreas after its exposure to carbon dioxide at 15 MPa 75 °C for 24 h. The activity was measured using 1,2-*O*-dilauryl-*rac*-glycero-3-glutaric acid resorufin ester (DGGR) as substrate and the maximum achieved increase in the activity was by 760%. The influence of the temperature, pressure and humidity of SC-CO_2_ and that of the number of pressurization and depressurization steps on the catalytic activity and stability of enzymes was examined in [44]. The treated enzymes were crude and a purified preparation of esterase EP10 from *Burkholderia gladioli*, lipase from *Candida rugosa* and esterase from porcine liver. An increase in enzyme activity was observed only for the crude preparation from *B. gladioli*; after 30 pressurization/depressurization cycles its activity increased by 20%. The increase might be connected with enzyme purity; the impurities that decreased the stability of the crude enzyme were extracted with CO_2_ that was introduced into the vessel containing the enzyme in each pressurization step. Fluorescence spectra indicated no conformational change before and after the treatment with SC-CO_2_.

Bauer *et al*. [45] studied the effects of a humid SC-CO_2_ medium on the activity of crude porcine pancreas lipase. Different substrates such as 1,2-*O*-dilauryl-*rac*-glycero-3-glutaric acid-resorufin ester (DGGR), triolein, tributyrin and triacetin were used in an enzyme assay. The treated preparations were found to be more active with a long chain triglyceride, e.g., triolein (the maximum residual activity was 675%) and DGGR, whereas there was a loss of activity towards short chain triglycerides, e.g., tributyrin and triacetin.

Independently, Yan *et al*. [46] measured the hydrolytic activity of lipase from *Candida rugosa* treated with SC-CO_2_. The activity increased approximately 2.5 times after the incubation for one hour at 35 °C and 19.6 MPa. Changes in the content of proteins in the lipase and in the size of the lipase were investigated using fluorescence emission spectroscopy and scanning electron microscopy, respectively. Both size reduction of lipase particles and the purification of enzyme might be the main reasons for the increase in the lipase activity.

The activity of lipases from porcine pancreas, *Candida antartica* recombinant from *Aspergillus oryzae*, *Candida cylindracea* (immobilized), *Penicilium roqueforti*, *Aspergillus niger*, *Rhizopus arrhizus*, *Mucor miehei* (two types of immobilization), *Pseudomonas cepacia* (two types of immobilization) were studied after using them as biocatalysts of blackcurrant oil hydrolysis under SC-CO_2_ conditions [47]. The reaction was performed at 40 °C and 15 MPa in a continuous-flow reactor. An increase of the relative activity of all used lipases after the hydrolytic reaction was observed. The most remarkable increase in the activity was observed for the lipase from *Rhizopus arrhizus* the activity of which increased more than 50 times. The highest activity was shown by Lipozyme^®^, lipase from *Mucor miehei* immobilized on macroporous resin. Both treated and untreated Lipozyme^®^ were used as biocatalysts in a hydrolytic resolution of racemic *cis*- or *trans*-isomers of 2-(4-methoxy-benzyl)cyclohexyl acetates. Satisfactory reaction yields (40%) and excellent enantiomeric purity of the products (E = 472) were obtained when hydrolysis of *trans*-isomer of 2-(4-methoxybenzyl)cyclohexyl acetate was catalyzed by Lipozyme^®^ treated with SC-CO_2_.

SC-CO_2_ has thus been successfully employed in a variety of applications due to its numerous advantages. Despite extensive investigations on the relationship between the activity of enzymes treated with supercritical fluids and supercritical operating conditions, there are no experimental studies that have addressed the effects of supercritical pretreatment on enzyme denaturation. The impact of SC-CO_2_ pretreatment on the activity and stability of hen egg-white lysozyme during its course of denaturation was also explored [48]. The data indicated no noticeable enhancement in the enzyme activity and stability in the presence of SC-CO_2_ pretreatment for lysozyme samples denatured by 8 M urea at 50 °C and pH 6.2. However, SC-CO_2_ pretreated lysozyme samples in 0.067 M phosphate buffer containing dithiothreitol (0.1 M dithiothreitol, pH 6.2, 25 °C or 0.01 M dithiothreitol, pH 6.2, 50 °C) at 17.24 MPa and 50 °C had better residual activity relative to samples that were not pretreated. In addition, when denaturing at 65 °C and pH 9.0, the pretreatment in SC-CO_2_ at 17.24 MPa and 50 °C resulted in the best stability of lysozyme. The result of this study may provide supporting evidence that supercritical fluids serve as potential media for enhancing the activity of enzymes used in a variety of biochemical applications.

l-Amino acid oxidase (l-AAO) from snake (*Crotalus adamanteus*) venom was successfully tested as a catalyst in SC-CO_2_ [49]. The enzyme activity was measured before and after its exposure to the supercritical conditions (40 °C and 11.0 MPa). It was found that l-AAO activity increased slightly by up to 15% after SC-CO_2_ exposure. l-AAO was more stable in SC-CO_2_ than in phosphate buffer under atmospheric pressure, as well as in the enzyme membrane reactor (EMR) experiment. 3,4-Dihydroxyphenyl-l-alanine (l-DOPA) oxidation was performed in a batch reactor made of stainless steel that could withstand the pressures of SC-CO_2_, in which l-amino acid oxidase from *C. adamanteus* was able to catalyze the reaction of oxidative deamination of l-DOPA in SC-CO_2_. For the comparison l-DOPA oxidation was performed in the EMR at 40 °C and pressure of 0.25 MPa. Productivity expressed as mmols of converted l-DOPA after 3 h per change of the enzyme activity after 3 h was the highest in SC-CO_2_ (1.474 mmol U^−1^), in which catalase was present, and the lowest in the EMR (0.457 mmol U^−1^).

The thermal stability and activity of enzymes in SC-CO_2_ and near-critical propane were studied at a pressure of 30.0 MPa in the temperature range 20–90 °C [42]. Lipases from *Pseudomonas fluorescences*, *Rhizopus javanicus*, *Rhizopus niveus* and porcine pancreas were found to be stable in SC-CO_2_ and near-critical propane (30.0 MPa, 40 °C) in their native form, and there was no activity change for any of the examined lipases.

An objective for the stability studies consisted in determining inactivation effects on lipase when exposed in SC-CO_2_ and in ionic liquids. After being exposed in the chosen solvent for 24 h at 60 °C, the lipase was used as biocatalyst for the synthesis of citronellyl laurate in *n*-heptane at 60 °C at atmospheric pressure [50].

Almost no difference between the synthetic processes catalyzed by the untreated lipase and by the lipase that was previously exposed to SC-CO_2_ and to [bmim][BF_4_] was observed. A residual activity of lipase that was previously exposed to SC-CO_2_ slightly decreased what resulted in lower ester concentration in first hour of the reaction performance. A decreased lipase residual activity can be explained by extraction of water essential for the enzyme from its vicinity by the SC-CO_2_, what was confirmed by the Karl–Fischer method. The untreated lipase contained 1.44% water, while the lipase treated with SC-CO_2_ contained only 0.88% water. Similar results were obtained when esterification was catalyzed by lipase that was previously exposed to bmim][BF_4_] and SC-CO_2_, whereas lower initial rates were obtained when the synthesis was catalyzed by a lipase treated with [bmim][BF_4_] compared to the synthesis catalyzed by the untreated lipase. The results indicated that the lipase was partially inactivated after being incubated in [bmim][PF_6_]. Visual observations showed that ionic liquids formed a strong ionic matrix and enzyme could be considered as being included into the media. Therefore, interactions of substrates with the active site of the lipase were limited resulting in lower ester concentrations. On the contrary, higher stability of *C. antarctica* lipase B and esterase from *B. stearothermophilus* in [bmim][PF_6_] compared to the organic solvents were reported [51,52].

Changes in activity between the crude enzyme preparation and the enzyme preincubated in SC-CO_2_ were, on one hand, connected with thermal activation/deactivation and, on the other hand, with water distribution in the system [53]. At higher temperatures water was “extracted” from the enzyme microenvironment by the SC-CO_2_, and this was the reason for the lower optimum temperature than at atmospheric pressure. Enzymes need a specific amount of a bound water to be active. This is of vital importance in biocatalysis in non-aqueous media. If the water content of carbon dioxide is too high or if water is a product in the reaction, the humidity can increase to a value where the enzyme is inactivated. SC-CO_2_ may dissolve from 0.3 to 0.5% (w/w) water, depending on the pressure and temperature [53].

## 4. Enzymatic Reactions in SC-CO_2_

### 4.1. Hydrolysis

There are only a few reports in the literature on enzymatic hydrolysis of vegetable oil or triacylglycerol in supercritical carbon dioxide in a flow-through reactor. Hampson and Foglia [54] measured the extent of conversion of tripalmitin to palmitic acid in SC-CO_2_ flowing through a bed of immobilized lipase from *Candida antarctica*, and showed the importance of enzyme moisture on the activity. Rezaei and Temelli [55,56] studied a Lipozyme-catalyzed hydrolysis of canola oil as a model reaction to develop an on-line extraction-reaction process, where fatty oil is extracted from oilseeds and converted to other valuable products using SC-CO_2_. Sovová and Zarevúcka [57] studied the extent of conversion and possible Lipozyme specificity towards fatty acids in hydrolysis of blackcurrant oil with respect to its dependence on the pressure, temperature, carbon dioxide flow rate and moisture content, enzyme load and enzyme distribution in the reactor.

Zarevúcka *et al*. studied lipase hydrolysis of blackcurrant seed oil, which is rich in α- and γ-linolenic acid, which the human organism is unable to synthesize *de novo* [58]. Lipozyme, a lipase from *Mucor miehei*, immobilized on macroporous anionic resin [59,60] was used as a biocatalyst of the reaction. The reaction was performed in a continuous flow reactor at 10–28 MPa and 30–50 °C with carbon dioxide saturated with oil and water (55–100%) flowing up through the enzyme bed. The analysis of a product composition indicated unfavorable hydrodynamics with significant mixing in the reactor when solvent interstitial velocity was lower than 4 mL min^−1^, while above this velocity value the flow pattern was near to the plug flow. Lipase stability was high, with no activity reduction observed during a long-term experiment. The reaction rate was a function of the ratio of enzyme loaded to a solvent volumetric flow rate. A complete hydrolysis of oil was achieved in the experiments carried out with the enzyme load of 0.8 g and CO_2_ flow rate of 0.4–0.9 g min^−1^. The effects of pressure (10–25 MPa) and temperature (30–40 °C) on the reaction rate were small, and the effects of CO_2_ saturation with water and of enzyme distribution in the reactor were negligible. Lipozyme displayed specificity towards linolenic acids; the release of γ-linolenic acid was faster and that of γ-linolenic acid slower than the release of other constituent acids present in blackcurrant oil. The experimental system was also studied and analyzed by means of the HPLC-NMR hyphenated technique [61].

Because lipases were found to be stable in SC-CO_2_, a lipase-catalyzed hydrolysis of sunflower oil was performed in two kinds of high-pressure reactors in this medium, a high-pressure batch stirredtank reactor (HP BSTR [62]) and a high-pressure continuous flat-shape membrane reactor (HP CFSMR [63]), in this medium. The reaction rates of enzyme-catalyzed reactions were higher than those of the conventional reactions. Higher conversion was achieved for hydrolysis in the HP BSTR after 48 h, but the maximum conversion in the HP CFSMR was achieved after only 1 h. In both cases the reaction parameters such as pressure, amount of biocatalyst and flow rate of the substrates (in HP CFSMR) had to be optimized.

Primožič *et al*. [64] studied the thermodynamic and kinetic properties of the immobilized lipase from *Aspergillus niger* (Lipolase 100T), and used this enzyme for catalysis of hydrolysis of sunflower oil in supercritical carbon dioxide. The optimal concentration of lipase was 0.0714 g/mL of CO_2_–free reaction mixture, and the highest conversion of oleic acid (0.193 g/g of oil phase) and linolenic acid (0.586 g/g of oil phase) were obtained at 50 °C, 20 MPa, pH = 7, and an oil/buffer ratio of 1:1 (w/w).

The enzymic hydrolytic reaction in SC-CO_2_ as a reaction medium to make glucose from starch was investigated [65]. The reaction rate was enhanced at higher temperature and pressure, especially near the critical point of the CO_2_. α-Amylase and glucoamylase were found to be active in SC-CO_2_.

The major advantage of immobilized enzymes consisted in an easier separation from the product solution. Reusability studies of immobilized enzyme preparation were performed at atmospheric pressure and in a system CO_2_/H_2_O at pressure of 10 MPa. The immobilized cellulase was successfully reused for more than 20 times without any significant loss of activity. Thermal stability studies of immobilized and crude cellulase in SC-CO_2_ showed that the activity of immobilized cellulase preincubated in SC-CO_2_ was much higher than the activity of crude cellulase exposed in SC-CO_2_ at the same conditions. The residual activity of pre-incubated on aerogel immobilized cellulase at 110 °C was 172%.

The immobilization of cellulase in silica aerogel matrix has shown to be very efficient as it has improved the biocatalytic properties of cellulase such as activity, stability and its repeated application for hydrolysis of carboxymethyl cellulose [66]. An optimum temperature for the hydrolytic reaction performed at atmospheric pressure was found to be 40 °C with the optimum quantity of 40 g L^−1^ of the immobilized cellulase. The activity of the immobilized cellulase rose for 110% compared to the activity of native cellulase in the reaction performed at atmospheric pressure. The incubation of the immobilized enzyme in SC-CO_2_ (10.0 MPa and 35 °C) prior the hydrolytic reaction improved residual activity up to 461%. The immobilization of cellulase on aerogel matrix also improved the thermal stability of enzyme, as residual activity after incubation at 110 °C in SC-CO_2_ was still more than 150%. The same incubation of the native cellulase at 50 °C deactivated the enzyme as the residual activity decreased to 30%. The reason for higher relative enzyme activities in the case of using a highpressure system CO_2_/H_2_O as a reaction medium was high dispersion of the cellulase in the silica aerogel matrix. The immobilized cellulase could be well reused without any significant loss of activity for at least 15 reaction cycles at atmospheric pressure and at least 20 reaction cycles when a highpressure system CO_2_/H_2_O was used as a reaction medium.

Proteinases are useful for regioselective hydrolytic transformations. Since papain is one of the few enzymes for organic synthetic transformations that originate from plant sources (the papaya in this case), proteinase from *Carica papaya* latex was tested for thermal stability at atmospheric pressure, as well as in SC-CO_2_ and near-critical propane [42]. The activity of proteinase from *Carica papaya* at atmospheric pressure increased with the temperature increase from 20 °C to 60 °C. The optimum temperature was determined to be 50 °C. The activity of the enzyme treated with SC-CO_2_ (30.0 MPa) also increased with increasing temperature, but the optimal temperature in this case was shifted to 40 °C.

### 4.2. Synthesis in SC-CO_2_

Many examples of biocatalytic synthesis have been documented, though few were optimized in a bioreactor in spite of their importance for large-scale preparation [67–70]. For widespread industrial use to occur, the process has to be technically and economically feasible. Batch vessels are suitable for preliminary screening of enzymic reactions, though, in order to obtain suitable reaction performance data for up-scaling, flow reactors are indicated. Among continuous reactors, packed-bed bioreactors have been extensively investigated for use in industrial scale applications. The packed-bed reactor (PBR) is one of the most commonly employed for solid–fluid contacting in heterogeneous catalysis, because: (i) it facilitates the contact and subsequent separation (ii) and the continuous removal of inhibitory substances; (iii) it allows reuse of the enzyme without the need of a prior separation; (iv) it permits to handle substrates of low solubility by using large volumes containing low concentrations of substrate; (v) it leads to more consistent product quality and improved enzyme stability due to the ease of automation and control [71]; (vi) it is suitable for long-term and industrial-scale production, differently from a stirred-tank reactor where enzyme granules would be susceptible to breakage because of the mechanical shear stress; and (vii) it is more cost effective than the batch operation [72–74]. The ratio between substrate and enzyme is much lower in a PBR than in conventional batch reactors, and it results in higher reaction performance. A stirred tank reactor has to be emptied and refilled at the end of the batch run, leading to downtime and a loss of productivity and, in addition, is subject to suffer from batch-to batch variations. Thus, in a PBR, a purer and more reproducible product and a far greater productivity from a fixed amount of enzyme than the one achieved in the batch process may be expected.

Furthermore, the easily tunable solvating power of SC-CO_2_ facilitates a relatively easy separation of reactants, products, and catalysts after reaction, whilst eventual separation of the reaction product from organic solvent is costly and time consuming and, thus, not suitable for industrial production. Furthermore, the high volatility of CO_2_ allows it to be completely and easily removed from the product, resulting in an overall solvent-free reaction, required for industrial manufacture, cosmetic and pharmaceutical applications, as *n*-octyl oleate uses.

At present, the main disadvantage of the use of enzymes in industrial applications is their cost. To overcome this problem, the enzyme is employed in immobilized form because it would allow the reutilization of the enzyme in continuous process [75]. Immobilized enzyme preparations exhibit easy recovering and recycling for subsequent reuse and high biocatalytic density, and offer economic benefits in terms of being able to produce a great quantity of product per unit of enzyme consumed [76] with sufficient operational stability for industrial application.

Capsaicin (8-methyl-*N*-vanillyl-6-nonenamide) is the main pungent component in capsicum fruits. Its analog, palmitoyl vanillylamide, with similar properties, was synthesized through amidation by lipase in SC-CO_2_. [55]. Among five lipases tested, immobilized *Mucor miehei* lipase, Lipozyme IM, was the most effective in catalyzing a synthesis of this analog. The reaction conditions for the analog synthesis were optimized at 50 °C, 17 MPa and pH 8, and the reaction proceeded for 23 h using vanillylamine hydrochloride and palmitic anhydride at a molar ratio of 5/15 as substrates. The reaction was catalyzed by Lipozyme IM at a concentration of 0.5% (w/w). The residual enzyme activity was about 40% and 15% after a 46- and 69-h repeated amidation reaction in a batch reaction under the optimized conditions, suggesting further modification of conditions was required.

Pyrrole was converted to pyrrole-2-carboxylate in SC-CO_2_ using cells of *Bacillus megaterium* PYR 2910, and the yield of the carboxylation reaction in SC-CO_2_ was 12 times higher than that under atmospheric pressure [78]. The cells of *Bacillus megaterium* PYR2910 [79–82] were employed for the CO_2_ fixation reaction. The reaction was conducted by adding CO_2_ to 10 MPa to the mixture of pyrrole, the cells, KHCO_3_, and NH_4_OAc in potassium phosphate buffer. For the reaction at the atmospheric pressure (0.1 MPa), the evolved CO_2_ was released to keep the pressure atmospheric. However, the yield was much higher for the reaction proceeding in supercritical CO_2_ than at atmospheric pressure. It was also confirmed by a control experiment without the cells that the non-biocatalytic carboxylation of pyrrole did not proceed. Therefore, the cell is surely catalyzing the CO_2_ fixation reaction more effectively in SC-CO_2_ than at atmospheric pressure.

The activity and stability of a cross-linked enzyme aggregate of *Candida antarctica* lipase B (CLEA-Calb) in SC-CO_2_ have been studied by Dijkstra *et al*. [83]. The model reaction used is the esterification of isoamyl alcohol with acetic acid. The catalytic performance of CLEA-Calb is evaluated both in batch and in continuous experiments, with a focus on the effect of water production upon reaction. The results of the batch experiments show a decreasing initial activity of the CLEACalb with an increase in pressure. Moreover, CLEAs appear to be highly stable in SC-CO_2_ and also remain active during continuous reactions.

Isoamyl acetate was successfully synthesized from isoamyl alcohol in SC-CO_2_ by two different immobilized lipases (Novozym 435 from *Candica antarctica* and Lipozyme RM-IM from *Rhizopus miehei*) [84]. Among several tested reactants, including acetic acid and two different acetates, acetic anhydride gave best yields. An esterification extent of 100% was obtained in continuous operation using acetic anhydride as acyl donor and Novozym 435 as enzyme.

Long-chain fatty acid esters are useful functional molecules responding to the requirements of numerous fields of application in cosmetic, pharmaceutical and lubricant industry. Lipase-catalyzed production of *n*-octyl oleate by esterification of oleic acid with 1-octanol in dense CO_2_, as reaction medium, was performed in bench-scale packed-bed bioreactor, in order to obtain suitable reaction performance data for up-scaling [85]. Lipase from *Rhizomucor miehei* (Lipozyme RM IM) was used as the biocatalyst. The experiments were planned to elucidate the effect of several process parameters, such as pressure, temperature, CO_2_ and substrates flow rates. Pressure of 10 MPa, temperature of 50 °C, CO_2_ flow rate of 210 L h^−1^ and substrate flow rate of 18 mL h^−1^ were predicted to be the optimum conditions: a maximum yield of about 93% was achieved. Performing the enzymic reaction in the continuously operating bioreactor, a long-term enzyme lifetime was observed and no decrease of the Lipozyme activity was registered over 50 days. A comparison with the experimental results obtained in a batch-wise mode was also proposed. Operating at the optimum reaction conditions, a higher ester yield than that obtained in batch-mode was detected. SC-CO_2_ was shown to be a potential medium for the biosynthesis of *n*-octyl oleate for a large-scale continuous-mode production.

Cutinase from *Fusarium solani pisi* was encapsulated in sol-gel matrices prepared with a combination of alkyl-alkoxysilane precursors of different chain-lengths [86]. The specific activity of cutinase in a model transesterification reaction at a fixed water activity in *n*-hexane was the highest for the precursor combination tetramethoxysilane/*n*-butyltrimetoxysilane (TMOS/BTMS) in a 1:5 ratio, lower and higher chain lengths of the mono-alkylated precursor or decreasing proportions of the latter relative to TMOS leading to lower enzyme activity. The behavior of the gels in SC-CO_2_ paralleled that exhibited in *n*-hexane, although cutinase activity was one order of magnitude lower (*i.e*., sol-gel encapsulation did not prevent the deleterious effect of CO_2_) The impact that functionalization of some of the additives had on cutinase activity indicates that the enzyme/matrix interactions play an important role. Some of the best additives from the standpoint of enzyme activity were also the best from the standpoint of its operational stability (ca. 80% retention of enzyme activity at the tenth reutilization cycle). None of the additives that proved effective for cutinase could improve the catalytic activity of sol-gel encapsulated *Pseudomonas cepacia* lipase.

The comparative study on the activity of *Fusarium solani pisi* cutinase immobilized on zeolites NaA and NaY, in *n*-hexane, acetonitrile, supercritical ethane (SC-ethane) and SC-CO_2_, at two different water activity (a_W_) values set by salt hydrate pairs in situ and at acid-base conditions fixed with solidstate buffers of aqueous pK_a_ between 4.3 and 10.6 was performed [87]. The reaction studied was the transesterification of vinyl butyrate by (*R*,*S*)-2-phenyl-l-propanol. The transesterification activity of cutinase was highest and similar in SC-ethane and in *n*-hexane, about one order of magnitude lower in acetonitrile and even lower in SC-CO_2_. Activity coefficients (γ) [88–90] generated for the two substrates indicated that they were better solvated in acetonitrile and thus less available for binding at the active site than in the other three solvents. The γ data also suggested higher reaction rates in SC-ethane than in *n*-hexane, as observed, and provided an evidence for a direct negative effect of SC-CO_2_ on enzyme activity. Manipulation of the acid-base conditions of the media did not afford any improvement of the initial rates of transesterification relative to the blanks (no added acid-base buffer, only salt hydrate pair), except in the case of cutinase immobilized on zeolite NaA in SC-ethane at a_W_ = 0.7. A poor performance of the blank in this case and a great improvement observed in the presence of a basic buffer suggest a deleterious acidic effect in the medium which, an experiment without additives confirmed, was not due to the known acidic character of the salt hydrate pair used to set a_W_ = 0.7. In acetonitrile, increasing a_W_ was accompanied by a decrease in initial rates of transesterification, unlike in the other solvents. There was a considerable hydrolysis in acetonitrile, where initial rates of hydrolysis increased about 20-fold from a_W_ = 0.2 to 0.7. The hydrolysis was less pronounced in SC-ethane and in *n*-hexane, and only at a_W_ = 0.7, and in SC-CO_2_ butyric acid was detected only at very long reaction times, in agreement with a generally low catalytic activity. Cutinase enantioselectivity towards the alcohol substrate was low and unaffected by any manipulation of medium conditions.

The catalytic activities of cutinase immobilized on zeolite NaY and *Candida antarctica* lipase B immobilized on an acrylic resin (Novozym 453) were measured in a model transesterification reaction in three imidazolium cation-based ionic liquids (RTILs), SC-ethane, SC-CO_2_ and *n*-hexane, at a water activity a_W_ of 0.2 and 0.7 [91]. The transesterification activity of cutinase was the highest and similar in 1-*n*-butyl-3-methylimidazolium hexafluorophosphate ([C(4)mim][PF_6_]), SC-ethane and *n*-hexane, more than one order of magnitude lower in SC-CO_2_, and increased with an increase in a_W_. Hydrolysis was not detected in SC-fluids and *n*-hexane, and was observed in RTILs at a_W_ 0.7 only. Both initial rates of transesterification and of hydrolysis of Novozym decreased with an increase in a_W_. SC-CO_2_ did not have a deleterious effect on Novozym activity, which was as high as in SC-ethane and *n*-hexane. The low reaction rates obtained in this case in RTILs suggested the existence of internal diffusion limitations absent in the cutinase preparation in which the enzyme is only adsorbed at the surface of the support. SC-CO_2_ did not adversely affect the catalytic activity of cutinase suspended in [C(4)mim][PF_6_], suggesting a protective effect of the RTIL. In the case of Novozym, remarkable increase in the rate of transesterification was obtained in the [C(4)mim][PF_6_]/SC-CO_2_ system, compared to the RTIL alone. This may reflect improved mass transfer of solutes to the pores of the immobilization matrix due to a high concentration of dissolved CO_2_ and a reduction in viscosity of the RTIL. Immobilized *Candida antarctica* lipase B was used as catalyst to synthesize butyl butanoate from vinyl butanoate and 1-butanol in SC-CO_2_ with excellent results [92]. The catalytic behavior of the enzyme immobilized on an acrylic support has been studied tank reactor, showing that a decrease in both the water content and the SC-CO_2_ density enhanced the synthetic activity and selectivity.

Polyphenolic compounds, such as caffeic acid, offer many health benefits, but their industrial applications are limited because of their low solubility in water and instability under UV light or heat. In the study of Shin *et al.* [93] caffeic acid glucosides were produced under enzymic catalysis, in both aqueous buffer and aqueous-supercritical carbon dioxide media, using a recombinant sucrose phosphorylase (SPase) from *Bifidobacterium longum*. Under SC-CO_2_, the amounts of the reaction products from the enzyme reaction were smaller than those in the aqueous reaction medium. However, this is the first report of the transglucosylation of caffeic acid by SPase, and also the first enzymic reaction with phenolic compounds conducted in a SC-CO_2_ phase.

The esterification reaction of geraniol with acetic acid catalyzed by Novozym was studied in SC-ethane and in SC-CO_2_ [94]. Water activity a_W_ had a very strong effect on enzyme activity, with reaction rates increasing up to a_W_ = 0.25 and then decreasing for higher a_W_. Salt hydrate pairs could not prevent changes in a_W_ during the course of reaction but were able to control a_W_ to some extent and had a beneficial effect on both initial rates of esterification and conversion in SC-ethane. The enzyme was more active in SC-ethane than in SC-CO_2_, confirming the deleterious effect of the latter already observed with some enzymes. The temperatures between 40 and 60 °C did not have a strong effect on initial rates of esterification, although reaction progress declined considerably in that temperature range. For the mixture of 50 mM acetic acid plus 200 mM geraniol, a 100% conversion was achieved at a reaction time of 10 h at 40 °C, 10.0 MPa, an a_W_ of incubation of 0.25, and a Novozym concentration of 0.55 mg mL^−1^ in SC-ethane. Conversion was below 50% in SC-CO_2_ at otherwise identical conditions. With an equimolar mixture of the two substrates (100 mM), 98% conversion was reached at 10 h of reaction in SC-ethane (73% conversion in SC-CO_2_).

The monoterpene lavandulol, an significant industrial component of the essential oil of plants of genus *Lavandula*, has been successfully converted to lavandulyl acetate by enzymatic catalysis in SC-CO_2_ using immobilized *Candida antarctica* lipase B (Novozym 435) [95]. The biotransformation was optimized with respect to substrate concentration, temperature and pressure/density. Conversion of up to 86% was observed at substrate concentration of 60 mM at 60 °C and 10 MPa. Increased temperature of the system resulted in lower enantioselectivity, whereas changes in pressure/density had little effect on this parameter. Immobilized *Candida antarctica* lipase B (Novozym 435) has been successfully used for number of esterification processes SC-CO_2_. Michor *et al*. showed that Novozym 435 may be used for the acetylation of menthol at temperatures up to 50 °C and pressure 10 MPa [96]. Enzymic esterification of hexanol [97], ibuprofen [98] and geraniol [99] have also been reported using Novozym 435 in SC-CO_2_, the last example employing acetic acid as acyl donor.

Among the various lipases, crude hog pancreas lipase is one of the cheapest commercial lipases available. Enzymic reactions using porcine pancreas lipase in SC-CO_2_ have been reported for enantioselective esterification of glycidol [100].

The esterification of palmitic acid with ethanol was studied at various temperatures (35 °C–70 °C) in the presence of three lipases (Novozym 435, Lipolase 100T and hog pancreas lipase) in SC-CO_2_ and under solvent-free conditions [101]. All enzymes showed an optimum temperature of 55 °C under both conditions. The conversion obtained in SC-CO_2_ and under solvent-free conditions with Novozym at optimal conditions was 74 and 97%, respectively. Although a higher apparent yield was obtained under the solvent-free conditions due to higher substrate and enzyme concentrations, the reaction in SC-CO_2_ is better because of lower enzyme loading, higher reaction rates and easier downstream processing.

Asymmetric reduction using alcohol dehydrogenases is usually conducted in aqueous media. There are often problems with solubility of substrates. Therefore, asymmetric reduction of various ketones using alcohol dehydrogenases was conducted in SC-CO_2_. Whole cells of *Geotrichum candidum* were used for reduction so that the addition of co-enzymes was avoided [102,103]. The reduction of *o*-fluoroacetophenone in SC-CO_2_ at 10 MPa was conducted using 2-propanol as reductant (hydrogen donor), which afforded (*S*)-1-(*o*-fluorophenyl)ethanol in 81% yield after 12 h. Additional substrates were used as substrates, and it was found that all of them were reduced by alcohol dehydrogenase in SC-CO_2_ with 2-propanol with a very high enantioselectivity (more then 99%).

Compared to the studies using hydrolytic enzymes in SC-CO_2_, dehydrogenase-catalyzed reactions in SC-CO_2_ have not yet been developed. Only two reports have been found for biocatalytic reduction in SC-CO_2_, and neither of these used flow reactors: asymmetric reduction using *Geotrichum candidum* [102], and reduction of butyroaldehyde by horse liver alcohol dehydrogenase with a fluorinated coenzyme [104]. The reduction of cyclohexanone was successful, and the biocatalyst was recycled up to four times with only a slight loss in activity. Recycling was not possible using the corresponding batch system because the biocatalysts cannot tolerate repeated depressurization at a very low temperature and separation of the product from the biocatalysts using organic solvents. This method was also applied for the asymmetric reduction of *o*-fluoroacetophenone, achieving excellent enantioselectivity (ee > 99%) and a higher space-time yield than the corresponding batch process.

The application of SC-CO_2_ as a solvent in enzyme-catalyzed reactions has been a matter of considerable research because of its favorable transport properties, which accelerate mass-transferlimited enzymic reactions [105]. The inherent gas-like low viscosities and high diffusivities of supercritical fluids increase the rates of mass transfer of the substrates to the enzyme. Conversely, the liquid-like densities of supercritical fluids result in higher dissolution compared to those observed for gases. Unlike the behavior of gases and liquids, the physical properties of supercritical fluids can be adjusted over a wide range by a relatively small change in pressure or in temperature [106]. Moreover, it might be relevant to stress much lower expenses of solvent in the supercritical medium compared with those of conventional solvents [107]. Another advantage of the use of supercritical fluids (gases) as reaction media is the simple separation of products of the reactions and water in a continuously operated reactor on an industrial scale, due to their different solubility in these media. Since the solvent strength of a supercritical fluid can be varied by changing pressure and temperature, the solubility of substances can easily be regulated. It can be done continuously at the outlet of the reactor, allowing an integrated reaction and separation step, and thus simplifying the downstream processing and recycling of the solvent [107]. Examples of industrially important hydrolytic processes catalyzed by lipases are productions of soaps [108], fatty alcohols [108] and monoacylglycerols [109,110]. Table 1 summarizes the examples of enzymic synthesis in SC-CO_2_ from the chapter.

## 5. Conclusions

The application of SC-CO_2_ and generally, the application of supercritical fluids–has become a modern, novel and advantageous technique for performing numbers of enzyme-catalyzed reactions. SC-CO_2_ has a big advantage over other supercritical fluids based on the gaseous nature of CO_2_ under conventional conditions. Any enzyme-catalyzed process performed in SC-CO_2_ may profit from the fact that the separation of the products from the reaction medium can easily be achieved by changing of the reaction conditions, *i.e.*, by changing pressure and temperature, at the output of the reactor. Subsequent re-use of the gaseous CO_2_ after its re-pressurization in the same reaction process represents another important factor which supports classification of the processes based on using SC-CO_2_ as reaction medium among “green” and sustainable technologies. Application of SC-CO_2_ medium for performing industrially important enzyme-catalyzed processes is going to become important “green” and sustainable, environmentally friendly and economical synthetic protocols.

## Figures and Tables

**Table 1.** Enzymatic synthesis in SC-CO_2_.

**Enzyme**	**Reaction**	**References**
Lipase from *Mucor miehei*	amidation	[77]
Cells of *Bacillus megaterium* PYR2910	carboxylation	[78–82]
Lipase from *Candida antarctica*	esterification	[83,95,97–99]
Lipase from *Candida antarctica*	acetylation	[96]
Lipase from *Rhizomucor miehei*	esterification	[85]
Cutinase from *Fusarium solani pisi*	transesterification	[86,87]
Porcine pancreas lipase	enantioselective	[100]
	esterification	
Novozym 435	esterification	[84]
Lipozyme RM-IM		
*Candida antarctica* lipase B	transesterification	[92,91]
Novozym 435	esterification	[101]
Lipolase 100T		
Hog pancreas lipase		
Sucrose phosphorylase from *Bifidobacterium longum*	transglycosylation	[93]
Novozym 435	esterification	[94]
Alcohol dehydrogenase from *Geotrichum candidum*	reduction	[102–104]
